# A randomized, single-dose, pharmacokinetic equivalence study comparing MB02 (proposed biosimilar) and reference bevacizumab in healthy Japanese male volunteers

**DOI:** 10.1007/s00280-021-04324-z

**Published:** 2021-07-16

**Authors:** Takashi Eto, Yuji Karasuyama, Verónica González, Ana Del Campo García

**Affiliations:** 1 Clinical Research Unit, Souseikai Hakata Clinic, Fukuoka, Japan; 2 Syneos Health Clinical K.K., Tokyo, Japan; 3 Medical Department, mAbxience Research S.L., Madrid, Spain

**Keywords:** MB02, Bevacizumab, Biosimilars, Pharmacokinetics, Safety, Japanese

## Abstract

**Purpose:**

MB02 is a biosimilar to bevacizumab that has demonstrated similar physicochemical and functional properties in in vitro studies to the reference bevacizumab (Avastin^®^). This study aims to assess the pharmacokinetic (PK) similarity of MB02 to the reference bevacizumab in Japanese population.

**Methods:**

This double-blind, randomized, parallel-group, single-dose PK study, was performed in healthy Japanese male volunteers. Subjects were equally randomized (1:1) to receive a single (3 mg/kg) IV dose of MB02 or reference bevacizumab. PK assessments were done up to 70 days post-dose. Non-compartmental parameters were calculated. PK similarity was determined using predefined equivalence range (0.80–1.25) for the area under the serum concentration–time curve from time 0 extrapolated to infinity (AUC_0–∞_). Immunogenicity samples were taken pre-dose and up to day 70. Safety was assessed throughout the study.

**Results:**

In total, 48 subjects (24 in each treatment group) were dosed. Consequently to the observed similar PK profile, the 90% confidence interval for the geometric means ratio for the primary PK endpoint, AUC_0–∞_, was within the predefined equivalence range (0.981–1.11). Forty-seven treatment-emergent adverse events (TEAEs) were reported in 20 subjects (41.7%) with comparable incidence among MB02 and reference bevacizumab groups (22 and 25, respectively), none of them was severe or serious. Anti-drug antibodies incidence was low and similar between treatment groups.

**Conclusions:**

Pharmacokinetic similarity of MB02 to reference bevacizumab was evidenced in Japanese healthy subjects, with comparable safety and immunogenicity profile between treatments. This study supports the biosimilarity of MB02 to reference bevacizumab in Japanese population. ClinicalTrials.gov identifier: NCT04238650.

## Introduction

Bevacizumab is a recombinant humanized immunoglobulin G1 monoclonal antibody that inhibits angiogenesis by binding to vascular endothelial growth factor (VEGF) and preventing its interaction with VEGF receptors on the surface of endothelial cells [1, 2]. Since its first worldwide approval in 2004, the use of the reference bevacizumab (Avastin^®^) still remains as part of the standard of care in the treatment of advanced cancers, observing consistent efficacy across indications and a well-established clinical efficacy and safety profile [3]. Bevacizumab can be considered the most extensively characterized anti-angiogenic treatment. However, access to this treatment option is limited by the high cost of the treatment, and, currently, the arrival of bevacizumab biosimilars may mitigate cost barriers for patients and increase access to such an important therapy in oncology [4]. Particularly, in Japan, biosimilars are approximately 30% cheaper than their reference products and can provide a solution for reducing national health-care costs [5].

In the development of biosimilars, or “Follow-on biological products” as designed in Japan, the guideline issued by the main National Health Authorities specifies that biosimilars should be highly similar to the already licensed original biologic as a reference product with respect to quality attributes, notwithstanding minor differences in clinically inactive component and, existing knowledge should be sufficiently predictive to ensure that any differences in this quality attributes have no adverse impact on the drug product or on its safety or efficacy [6–11]. The assessment of similarity must follow a stepwise approach with the demonstration of analytical and bio-functional similarity in nonclinical studies as the first step, followed by clinical studies comparing the proposed biosimilar with the reference product to verify the equivalence of pharmacokinetics (PK) and, if appropriate, pharmacodynamics as a second step. Finally, as the last step, a confirmatory clinical trial in a sensitive population of patients might be needed. Biosimilarity assessment of a clinical trial should be based on the available data to design and step-by-step, and the type and content of the required clinical trial will vary greatly depending on reference biological product information and characteristics. Where PK/PD studies are sufficient to assure biosimilarity in the clinical endpoint of interest, the aforementioned, additional clinical studies to evaluate efficacy might be omitted [9]. This should be addressed on a case-by-case basis based on data obtained at the developmental stage and should, therefore, be consulted with regulatory authorities.

MB02 was developed as a biosimilar to the reference product bevacizumab by mAbxience Research SL following the recommendations for biosimilar products [6–11]. As with their other biosimilar products, mAbxience has based the development of MB02 on a “built-in by-design” biosimilarity approach. Following the defined Quality Target Product Profile, which corresponds to a set of quantitative ranges for key quality attributes based on data collected on the reference product, mAbxience established the critical quality attributes for MB02, and was designed accordingly. Through an extensive physicochemical and functional characterization, which included primary structure, higher order structure, biological activity, and binding affinity to VEGF, MB02 has demonstrated a similar quality profile to the reference bevacizumab. Furthermore, PK similarity has been demonstrated in two equivalence studies comparing the PK profiles of MB02 and reference bevacizumab (US- or EU-approved) following the administration of a single dose (3 mg/kg IV) in 228 healthy Caucasian male volunteers (ClinicalTrials.gov: NCT04238663 and NCT03293654). Recently, as the last step in clinical development of MB02 to contribute to the totality of evidence, similar efficacy and safety has been demonstrated in a pivotal clinical equivalence study in 627 patients with non-squamous non-small cell lung cancer (NSCLC) (ClinicalTrials.gov NCT03296163).

As part of the global clinical development for MB02, the current supportive PK equivalence study aims to provide evidence for PK similarity of the bevacizumab biosimilar MB02 by comparing the PK profiles of MB02 with EU-approved reference bevacizumab in a population of healthy Japanese male volunteers. The safety and immunogenicity of MB02 and reference bevacizumab were also compared in the study.

## Subjects and methods

### Study population and design

This double-blind, randomized, parallel-group, single-dose, two-treatment group study was performed in one center located in Fukuoka, Japan. (NCT04238650) The study was conducted in compliance with the ethical principles of the Declaration of Helsinki, International Council for Harmonization (ICH) Good Clinical Practice Guidelines (E6), and local regulatory requirements. Ethical approval for final protocol, any amendments, and informed consent documentation was sought and granted by Hakata Clinic Institutional Review Board. All subjects provided written informed consent before any study-specific procedures were performed.

Japanese healthy male subjects aged 20–55 years, with a total body weight of 50–100 kg and a body mass index (BMI) of 18.5–28 kg/m^2^ were eligible to be included in the study. Subjects must have no clinically significant findings from medical history, physical examination, 12-lead electrocardiogram (ECG), vital signs and clinical laboratory tests at screening. Key exclusion criteria included clinically significant essential hypertension, orthostatic hypotension, cardiac failure or history of thromboembolic conditions or other clinically significant disease; significant hypersensitivity, intolerance or allergy to any drug compound; previous treatment with other antibody or protein targeting VEGF or VEGF receptor.

### Procedures and outcomes

Subjects that were considered eligible at the screening visit (within 28 days before dosing) were admitted to the Clinical Research Unit (CRU) on Day 1 (day before dosing) to recheck eligibility, and those who were administered MB02 or reference bevacizumab were confined at the CRU until discharge on Day 8. On Day 1, eligible subjects were stratified into two groups based on their body weight (stratum 1: ≥ 50 to < 67 kg and stratum 2: ≥ 67 to ≤ 100 kg) and were randomized (1:1 ratio) to receive a single dose of 3 mg/kg of MB02 or reference bevacizumab by IV infusion over 90 min. Randomization was performed using a computer-generated pseudo random permutation procedure provided by an unblinded statistical team from a third-party provider. All subjects involved into the study (investigators, nurses, or sponsor), except of the pharmacist in charge of the treatment preparation, were blinded to the assigned treatment until the end of study.

Subjects were discharged on Day 8 and returned on Days 10, 14, 21, 28, 38, 50, 62 and 70 for non-residential visits for the collection of PK and immunogenicity samples (when applicable) and for safety assessments. Subjects were monitored for adverse events and treatment-emergent adverse events (TEAEs) on an ongoing basis throughout the study until the end of study (Day 70). TEAEs were defined as those adverse events that occurred after study drug administration or that pre-existed and worsened in severity after study drug administration. Subjects were followed up until any unresolved adverse event or its sequelae resolved or stabilized per the investigator assessment. Concomitant medication use was also recorded during the study. Other safety assessments included clinical laboratory tests (hematology, biochemistry, coagulation, and urinalysis), vital signs, 12-lead ECG, and physical examination.

### Sampling and bioanalytical methods

Blood samples for PK sampling were collected by venipuncture or cannulation at time 0 (pre-dose), end of infusion, 2, 3, 4, 5, 6, 8, 12 and 24 h after start of infusion, on Day 3–8 at CRU and on the day of the scheduled non-residential visits.

The analysis of serum drug concentrations (MB02 and reference bevacizumab) were performed at a qualified laboratory, using a validated enzyme-linked immunosorbent assay (ELISA) method.

Briefly, VEGF was coated on a 96-well microtiter plate, and then blocked using a nonspecific protein. MB02 was used to prepare standards and quality controls, this was then added to designated sample wells. The assay was visualized by the subsequent additions of anti-Human IgG1-HRP and a chromogenic substrate (3,3′,5,5′-tetramethylbenzidine), and the product of this reaction was detected with a spectrophotometer (450 nm detection and 630 nm reference wavelengths). The concentration of MB02 or reference bevacizumab in samples was then back-calculated from a MB02 calibration curve. Samples, standards and controls were required to be subjected to a minimum required dilution of 1 in 10 in Low Cross Buffer prior to analysis. Calibration curve fit: 4-PL weighted (1/Y2). The assay measured free drug concentrations in human serum, and the lower limit of quantification (LLOQ) for the method was 400.00 ng/mL.

Acceptable inter-assay precision (IAP) and inter-assay accuracy (IAA) were calculated from quality controls (QCs, LLOQ, low-, medium- and high-quality control sample and upper limit of quantification as well) in 12 validation runs for MB02 (IAP ≤ 6.2% and IAA ≤  + 13.1%), and in six validation runs for EU-bevacizumab (IAP ≤ 7.6% and IAA ≤  + 6.3%). The performance of the method during the sample analysis study was also acceptable (IAP ≤ 13.7% and IAA ≤  + 5.2%). Incurred sample reanalysis demonstrated reproducibility of drug concentrations in study samples.

The immunogenicity of MB02 and EU-bevacizumab were determined by detections of anti-MB02 and anti-reference bevacizumab antibodies in serum. The blood samples were collected by venipuncture or cannulation at the Day 1 and post-dose samples for ADA and NAb, collected at Days 14, 28, 56, 78 to determine the incidence of treatment-induced antibodies. A validated semi-quantitative immunoassay was used for the detection, confirmation, and titration of anti-MB02 and anti-bevacizumab antibodies in human serum samples collected from study subjects. The data were generated using Meso Scale Discovery [MSD; electrochemiluminescence (ECL)] platform. The immune response was evaluated by a three-tiered approach which compromised an immunogenicity assay for the screening, confirmation, and titration. All samples were subjected to an initial screening assay (Tier 1), and those falling above a specific pre-determined screening cut-point were tested in the confirmation assay (Tier 2). Samples that confirmed positive in the confirmatory assay were deemed positive and further analyzed in the titer tier (Tier 3), and for the presence of neutralizing antibodies.

The ADA assay used an ADA bridging format with acid dissociation. The ADA/drug complexes were acid dissociated to release any anti-bevacizumab antibodies complexed with free drug, which were then neutralized with neutralization buffer containing VEGF R1 to mitigate VEGF interference, and captured with biotinylated and sulfo-tagged MB02-labelled material. The antibody-bridge complexes were bound to a streptavidin-coated plate, and the chemiluminescent signal was read on an MSD ECL platform. Assay sensitivity was 20.0 ng/mL (without drug, low positive control) with a drug tolerance of 200.0 μg/mL at 100.0 ng/mL ADA. The overall inter-assay precision for positive control samples was ≤ 12.4%.

A validated qualitative ligand binding assay was used to detect neutralizing anti-MB02/reference bevacizumab antibodies in human serum using streptavidin magnetic beads and read on the MSD ECL platform. The signal produced was inversely proportional to the concentration of neutralizing anti-MB02/anti-bevacizumab antibodies present.

Additionally, the influence of ADA on PK was analyzed.

### Study objectives and endpoints

The primary objective of the study was to demonstrate PK similarity, assessed by comparing the area under the concentration–time curve extrapolated to infinity (AUC_0–∞_), between MB02 and reference bevacizumab. Secondary endpoints included derived PK parameters not covered by the primary PK endpoint, including but not limited to, the maximum observed serum concentration (C_max_), the AUC from time 0 to last observable concentration (AUC_0–t_), the time of maximum observed serum concentration (t_max_), total body clearance of drug after IV administration (CL), and serum terminal elimination half-life (t_1/2_).

The safety (including immunogenicity) of MB02 and reference bevacizumab were compared as secondary objectives of the study.

### Statistical methods

PK equivalence was demonstrated if the 90% confidence interval (CI) for the geometric least square means (GLSM) of the MB02:reference bevacizumab ratio for the primary endpoint, AUC_0–∞_, was fully contained within the predefined 0.80–1.25 equivalence range. The point estimate and 90% CIs for the ratio were assessed for AUC_0–∞_ and for the secondary endpoints C_max_ and AUC_0–t_ using an analysis of covariance (ANCOVA) on the natural-logarithmic-transformed PK parameters. The model included a fixed effect for treatment group and body weight as a covariate [12].

Study sample size was estimated assuming a coefficient of variation of 20% for AUC_0–∞_ following recent bevacizumab follow-on studies available and model-based simulations accounting intrinsic PK altering factors (body weight, gender, serum albumin and alkaline phosphatase), between-subject variability and residual variability [13–16] Assuming an expected true ratio of means between 0.95 and 1.05 for AUC_0–∞_ an estimated sample size of 48 subjects (considering a 5% drop-out rate) were required to achieve 90% power for the treatment group comparisons for the primary PK endpoint using a two-sided test at the 5% significance level.

Other secondary PK parameters as well as safety and immunogenicity were analyzed descriptively. PK analyses were performed in the PK population (all subjects receiving the full dose of MB02 or reference bevacizumab, did not have any major protocol deviations and have sufficient data to calculate primary PK endpoint and were not otherwise non-evaluable). Serum concentrations of bevacizumab were used to calculate the following PK parameters by standard non-compartmental methods using the validated software program, Phoenix WinNonlin^®^ (Certara USA, Inc. Version 8.1). Data analyses were performed using Statistical Analysis System (SAS^®^) Version 9.4.

Safety analyses were performed in all subjects receiving the study medication (safety population). Adverse events were coded and listed, by system organ class and preferred term, using the Medical Dictionary for Regulatory Activities (MedDRA version 22.1) and were summarized by severity (according to the National Cancer Institute Common Terminology Criteria for Adverse Events; NCI-CTCAE version 5.0), seriousness and relationship to study treatment. Clinical laboratory tests, physical examination, vital signs and ECG data were summarized by time point and treatment. A summary of the number and percent of subjects developing ADAs was presented for each treatment and overall.

## Results

### Study subjects

**Fig. 1:**
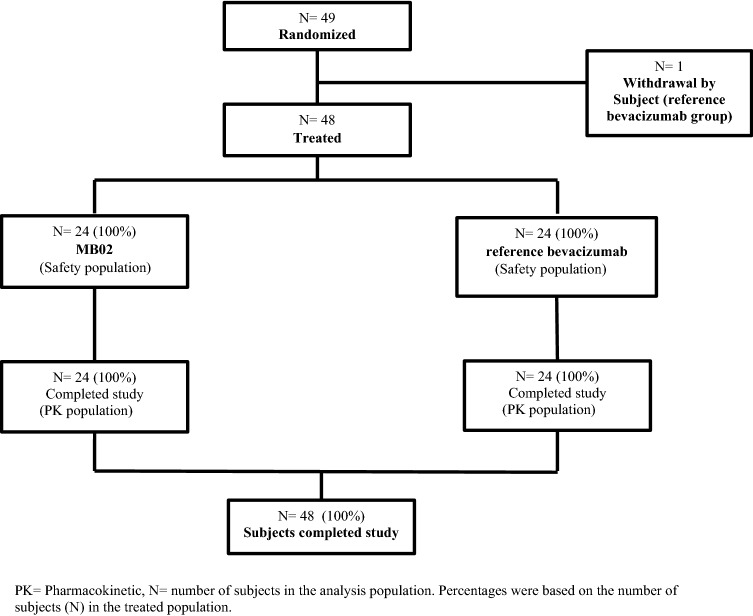
Study participants flow

The study was conducted between 2nd August 2019 and 27th December 2019. A total of 49 healthy Japanese male volunteers were selected and randomly assigned to MB02 (24 subjects) or reference bevacizumab (25 subjects). One subject assigned to reference bevacizumab group withdrew consent before receiving the study treatment. A total of 48 subjects (24 in each treatment group) received the study treatment, completed all the study procedures and were included in the safety and PK populations (Fig. 1). Subjects were all Japanese, most (95.8%) aged between 20 and 40 years. Demographic characteristics of study subjects were comparable between the treatment groups (Table 1).

**Table 1:** Baseline characteristics and demographics (Safety population)

Characteristics	MB02 *N* = 24 (%)	Reference bevacizumab *N* = 24 (%)
Race, *n* (%)		
Asian	24 (100)	24 (100)
Age (years)		
Mean (SD)	28.3 (5.9)	28.5 (6.6)
Height (cm)		
Mean (SD)	172.06 (6.31)	171.32 (6.27)
Weight (kg)		
Mean (SD)	67.00 (9.48)	68.50 (9.13)
≥ 50 to < 67 kg	59.17 (4.30)	61.36 (4.09)
≥ 67 to ≤ 100.0	74.83 (5.98)	75.63 (6.81)
BMI (kg/m^2^)		
Mean (SD)	22.54 (2.26)	23.33 (2.84)

BMI (kg/m^2^) = weight (kg)/(height [m])^2^. Percentages were based on the number of subjects (N) in the safety population

*BMI* body mass index, *N* number of subjects in the analysis population, *n* number of subjects within the category, *SD* standard deviation

### Pharmacokinetics

**Fig. 2:**
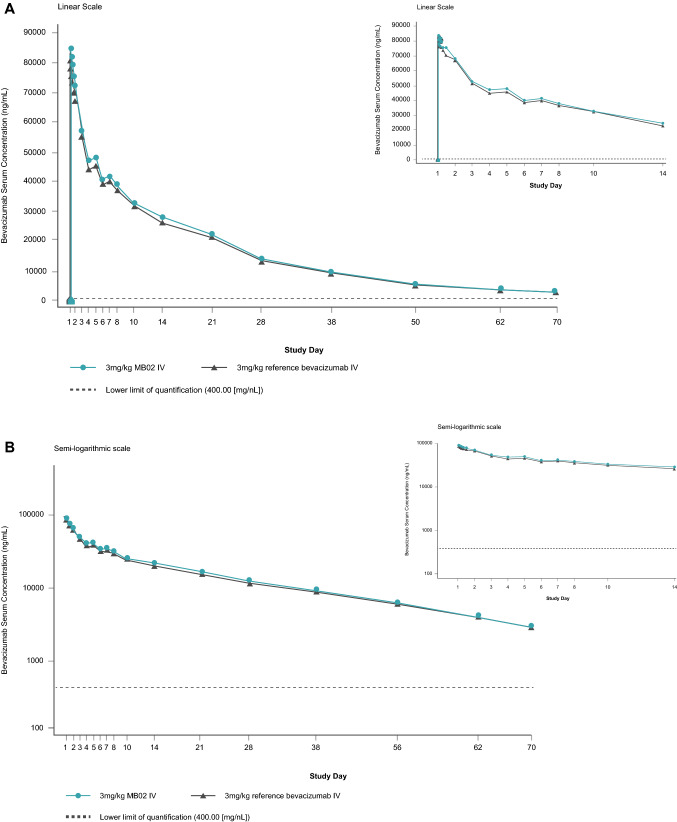
Arithmetic mean serum concentration–time profiles of bevacizumab following single IV doses of MB02 and reference bevacizumab to healthy Japanese male subjects (up to 14 days and across All days). Pharmacokinetic population [Linear (**A**) and Semi-Logarithmic (**B**) Scale]

Following IV infusion of a single 3 mg/kg dose of MB02 or reference bevacizumab to study subjects, the mean serum concentration–time profile observed in each treatment group was similar (Fig. 2).

The statistical analysis of the primary PK endpoint, AUC_0–∞_, and the secondary endpoints, C_max_ and AUC_0–t_, by treatment group was assessed for PK similarity (Table 2). For AUC_0–∞_, the ratio of GLSM (MB02 to reference bevacizumab) was 1.04 with 90% CI (0.98–1.11) fully contained within the predefined equivalence range of 0.80–1.25, confirming PK equivalence between MB02 and reference bevacizumab. The 90% CIs for the GLSM ratio of the secondary endpoints, C_max_ and AUC_0–t_, were also fully contained within the predefined equivalence range.

**Table 2:** Statistical analysis of pharmacokinetic endpoints (pharmacokinetic population)

Parameter (unit)	Treatment	*N*	Ratio of GLSM	90% CI of the ratio
AUC_0–∞_ (h ng/mL)	MB02	24	1.04	0.981–1.11
	Reference bevacizumab	24		
AUC_0–t_ (h ng/mL)	MB02	24	1.05	0.997–1.11
	Reference bevacizumab	24		
C_max_ (ng/mL)	MB02	24	1.13	1.03–1.24
	Reference bevacizumab	24		

The PK parameters were log transformed (base e) before analysis and analyzed using an analysis of covariance (ANCOVA) model. The model included treatment as a fixed effect and body weight as a covariate. The ratio and corresponding CIs were back-transformed from the difference and CIs calculated on the log_e_ scale

*AUC* area under the serum concentration–time curve; *AUC*_*0–∞*_ AUC from time 0 to infinity; *AUC*_*0–t*_ AUC from time 0 to last observable concentration; *CI* confidence interval, *C*_*max*_ maximum observed serum concentration

Furthermore, the comparison analysis of the individual PK parameters showed that MB02 PK and its associated variability were similar to the reference product (Table 3). Low between-subject variability was observed for MB02 and reference bevacizumab treatment groups with geometric CV% of AUC and C_max_ values ranging from 9.6 to 24.1%. For both treatment groups, the maximum observed serum concentrations of bevacizumab were delayed, with median t_max_ occurring between 4.0 and 4.5 h post the start of infusion. No trend was noted for t_max_ between treatment groups with similar median values and overlapping ranges. After reaching C_max_, observed serum concentrations declined in a biphasic manner. Similar geometric mean (%CV) elimination half-lives (t_1/2_) of 430 and 450 h were determined for MB02 and reference bevacizumab, respectively.

**Table 3:** Summary of pharmacokinetic parameters after a single IV dose of MB02 or EU-bevacizumab to healthy Japanese male volunteers (pharmacokinetic population)

Parameter	3 mg/kg MB02 IV *N* = 24 (%)		3 mg/kg reference bevacizumab IV *N* = 24 (%)	
	Geometric mean (geometric CV%)	Arithmetic mean (SD)	Geometric mean (geometric CV%)	Arithmetic mean (SD)
AUC_0–∞_ (h·ng/mL)	30,200,000 (14.9)	30,500,000 (4,640,000)	29,000,000 (11.1)	29,200,000 (3,270,000)
AUC_0–t_ (h·ng/mL)	28,100,000 (12.7)	28,300,000 (3,670,000)	26,900,000 (9.55)	27,000,000 (2,580,000)
%AUCextrap (%)	NA	6.77 (2.93)	NA	7.38 (2.58)
C_max_ (ng/mL)	92,400 (24.1)	95,200 (26,800)	82,800 (17.4)	83,900 (14,400)
t_1/2_ (h)	430 (16.4)	436 (73.1)	450 (12.9)	454 (57.2)
kel (1/h)	0.00161 (16.4)	0.00163 (0.000261)	0.00154 (12.9)	0.00155 (0.000204)
CL (L/h)	0.00660 (18.3)	0.00670 (0.00124)	0.00702 (14.4)	0.00709 (0.00111)
*V*_z_ (L)	4.10 (13.2)	4.13 (0.546)	4.56 (12.5)	4.59 (0.583)
*V*_ss_ (L)	3.88 (11.7)	3.91 (0.461)	4.28 (13.3)	4.32 (0.586)

	Median (minimum, maximum)	Median (minimum, maximum)
t_max_ (h)	4.50 (1.52, 24.0)	4.00 (1.52, 12.00)

*AUC* area under the serum concentration–time curve, *AUC*_*0–∞*_ AUC from time zero extrapolated to infinity, *AUC*_*0–t*_ AUC from time zero to the time of last quantifiable concentration, *CL* total body clearance of drug after intravenous administration, *C*_*max*_ maximum observed serum concentration, *CV* coefficient of variation, *IV* intravenous, *kel* elimination rate constant of the terminal phase, *mg* milligram, *N* number of subjects, *t*_*½*_ apparent serum terminal elimination half-life, *t*_*max*_ time of maximum observed serum concentration, *V*_*ss*_ volume of distribution at steady state after intravenous administration, *V*_*z*_ volume of distribution during the terminal elimination phase after intravenous administration, *%AUC*_*extrap*_ percentage of AUC that is due to extrapolation from the last quantifiable concentration to infinity

### Safety

Twenty (41.7%) of 48 subjects receiving a single dose of MB02 or reference bevacizumab reported TEAEs (47 in total). The proportion of subjects reporting TEAEs in the MB02 group was slightly lower (8 subjects; 33.3%) than in the reference bevacizumab (12 subjects; 50.0%); however, the number of TEAEs reported in each group was comparable (22 vs 25 TEAEs reported in subjects receiving MB02 or reference bevacizumab, respectively) (Table 4).

**Table 4:** Most frequent (reported in > 1 subject) treatment-emergent adverse events (safety population)

Adverse event by PT	MB02 *N* = 24 *n* (%) [E]	Reference bevacizumab *N* = 24 *n* (%) [E]	Overall *N* = 48 *n* (%) [E]
Subjects with at least 1 TEAE, *n* (%)	8 (33.3) [22]	12 (50.0) [25]	20 (41.7) [47]
Severity			
Mild	8 (33.3) [20]	9 (37.5) [17]	17 (35.4) [37]
Moderate	2 (8.3) [2]	6 (25.0) [8]	8 (16.7) [10]
Severe	0	0	0
Subjects with any related TEAE*	0	2 (8.3) [2]	2 (8.3) [2]
Subjects with any serious TEAE	0	0	0
Any TEAE			
Nasopharyngitis	2 (8.3) [2]	6 (25.0) [6]	8 (16.7) [8]
Pharyngitis	1 (4.2) [1]	4 (16.7) [5]	5 (10.4) [6]
Alanine aminotransferase increased	2 (8.3) [3]	2 (8.3) [2]	4 (8.3) [5]
Blood creatine phosphokinase increased	3 (12.5) [4]	1 (4.2) [1]	4 (8.3) [5]
Aspartate aminotransferase increased	2 (8.3) [3]	1 (4.2) [1]	3 (6.3) [4]
Blood lactate dehydrogenase increased	2 (8.3) [3]	0	2 (4.2) [3]

Percentages were based on the number of subjects in the safety population. If a subject had multiple events with different severity (or causality), then the subject was counted only once at the worst severity (or causality)

*TEAE* treatment-emergent adverse event, *E* number of TEAEs, N number of subjects in the analysis population, *n* number of subjects with event

*Considered related (possibly, probably or definitively related) to study drug by the investigator

All TEAEs were considered mild [37 TEAEs in 17 (35.4%) subjects] or moderate (10 TEAEs in 8 (16.7%) subjects) in severity, and incidences were comparable between treatment groups. No TEAE was rated as severe. The most common TEAEs were nasopharyngitis and pharyngitis, both of which occurred with higher frequency in reference bevacizumab treatment group.

Most TEAEs [44 (93.6%)] were considered not related to study treatment. TEAEs were considered as possibly related in two subjects receiving reference bevacizumab, a mild aphthous ulcer and mild abnormal hepatic function. Bevacizumab-related TEAEs (i.e., those commonly reported in the reference bevacizumab product information [3, 4]) were reported in one subject and mainly consisted in mild epistaxis that was considered unlikely related to reference bevacizumab. There were no serious AEs and no deaths or discontinuations occurred due to AEs in this study. Clinical laboratory data, vital signs, and 12-lead ECG parameters did not show any clinically relevant changes over time and no relevant differences between treatment groups.

### Immunogenicity

Forty-seven (97.9%) subjects tested negative for treatment-induced ADA (TI-ADA) at all timepoints. Only one subject tested positive for TI-ADA during the study following administration of reference bevacizumab. This positive TI-ADA result was transient, occurred at Day 28 and Day 50, and had low titers value (< 1) for both timepoints. TI-ADA in this subject had a NAb-positive response in both samples.

More importantly, the development of ADA or NAb responses was considered to have no effect on PK parameter estimates (clearance). The subject who developed an immune response to reference bevacizumab did not report any infusion reaction or any TEAE considered temporally associated with the development of the ADA or NAb.

## Discussion

Results from this single-dose comparative study confirm PK equivalence between MB02 and reference bevacizumab following a 3 mg/kg dose administered as a 90-min IV infusion in a population of healthy Japanese male volunteers. The study met the equivalence criteria as, for the primary endpoint (AUC_0–∞_), the 90% CI for the GLSM ratio (0.98–1.11) was fully contained within the predefined equivalence range of 0.80 to 1.25. In addition, for the secondary endpoints AUC_0–t_ and C_max_, the 90% CIs for the GLSM ratio MB02:reference bevacizumab were also fully contained within the predefined equivalence range, conferring robustness to PK similarity results. The serum concentration–time profiles for bevacizumab following IV infusion administration of 3 mg/kg of MB02, and reference bevacizumab were characterized by a biphasic decline in serum concentrations and, were comparable and consistent with known PK data available with reference bevacizumab [17].

The selected 3 mg/kg dose was well tolerated in all healthy Japanese volunteers, observing no remarkable differences between MB02 and reference bevacizumab. The incidence, severity and nature of TEAEs reported were comparable across treatment groups. No infusion reactions, severe or serious events or events leading to study discontinuation were reported and drug administration was not interrupted due to any TEAE. The safety profile reported for MB02 in this study is in line with that observed in previous studies with other bevacizumab biosimilar drugs in healthy Japanese male volunteers, and with the historical data available for reference bevacizumab [17, 18]. Immunogenicity profile for MB02 and reference bevacizumab was also comparable, observing a low incidence of TI-ADA (only one subject) and with no apparent effect on safety or PK profiles.

This study is part of the clinical package to seek approval of MB02 bevacizumab biosimilar in Japan. According to guidelines on biosimilars issued by Ministry of Health Labour and Welfare in Japan, a single-dose study was needed to be included in the clinical package to assess the safety and PK of the proposed biosimilar vs the reference product in either Japanese healthy subjects or patients to consider ethnic factors and, assess consistency with results in non-Japanese subjects [9–11, 19, 20].

As per international guidelines on biosimilarity, the assessment of PK in healthy volunteers is considered the most homogeneous and sensitive setting to detect differences in PK between MB02 and reference bevacizumab [6–9]. A study in healthy volunteers avoids factors that could confound the interpretation of PK, safety and tolerability results in patient studies, including varying tumor burden and complications arising from the disease state, comorbidities and concomitant therapies and medications. Bevacizumab PK is linear between 1 and 10 mg/kg, which allows dosing in healthy volunteers at lower doses than those indicated for therapeutic indications, reducing the risk of adverse events in healthy volunteers while still obtaining informative PK data [21]. For this reason, a dose of 3 mg/kg administered in a 90-min IV infusion was selected for the study as it balanced the safety considerations in healthy volunteers with the requirement to capture the full PK profile.

Other parameters such as gender and body weight are also known to affect bevacizumab PK, and for this end only male subjects with a BMI within 18.5–28 kg/m^2^ were enrolled and randomization was stratified by body weight [22, 23]. Results from a robust population PK model for bevacizumab published by Han K et al. included an assessment of ethnicity as covariate and did not identify a significant effect of ethnicity on PK [21]. The PK model was externally validated using 1670 serum bevacizumab concentrations from 146 Japanese patients; therefore, it is expected that non-Japanese and Japanese populations exhibit similar bevacizumab PK behavior.

Given that the results of the current study are consistent with the results of the two single-dose PK equivalence studies comparing MB02 with available sources of bevacizumab reference product (EU- and US-approved bevacizumab) in a non-Japanese population, there is cause to expect that results may be extrapolated to a Japanese population. (NCT04238663) Both studies met their primary objective to establish PK equivalence of MB02 and reference bevacizumab in terms of the primary endpoints AUC0–∞ and C_max_ and the secondary parameter AUC_0–t_, as the 90% CI for the GMLS ratios for the MB02 versus reference bevacizumab, MB02 versus US-approved bevacizumab and EU- versus US-approved bevacizumab comparisons were fully contained within the predefined equivalence range of 0.80–1.25. The safety and immunogenicity profile were also similar for all three treatment groups. Furthermore, considering that there is no a significant effect of ethnicity on PK this study allows to extrapolate the recent results from the pivotal clinical equivalence study in 627 NSCLC patients included in the global clinical package for MB02 (ClinicalTrials.gov NCT03296163) [24] to Japanese population.

In conclusion, the results from this PK study provide strong evidence supporting the PK similarity of MB02 to reference bevacizumab in healthy Japanese male volunteers, with comparable safety and immunogenicity profile between treatments. This study supports the biosimilarity of MB02 to reference bevacizumab in Japanese population. ClinicalTrials.gov identifier: NCT04238650.

## Data Availability

The data that support the findings of this study are available from the corresponding author upon reasonable request.
